# Assessing the experiences of intra-uterine device users in a long-term conflict setting: a qualitative study on the Thailand-Burma border

**DOI:** 10.1186/s13031-015-0034-9

**Published:** 2015-02-12

**Authors:** Jillian Gedeon, Saw Nanda Hsue, Meredith Walsh, Cari Sietstra, Hay MarSan, Angel M Foster

**Affiliations:** Faculty of Health Sciences, University of Ottawa, 1 Stewart Street, Room 312-B, Ottawa, ON K1N 6N5 Canada; Mae Tao Clinic, Mae Sot, Thailand; Cambridge Reproductive Health Consultants, Cambridge, MA USA; Ibis Reproductive Health, Cambridge, MA USA

**Keywords:** Myanmar, Contraception, Family planning, Abortion, Maternal health, Refugees

## Abstract

**Background:**

In Burma, severe human rights violations, civil conflict, and the persecution of ethnic and linguistic minority populations has resulted in the displacement of millions of people, many of whom now reside as internally displaced populations (IDPs) in Eastern Burma or in Thailand as refugees or undocumented migrants. Use of the intra-uterine device (IUD), a non-user dependent and highly reliable method of long acting reversible contraception, has the potential to make a significant impact on reproductive health in this protracted conflict setting.

**Objectives:**

This qualitative study aimed to understand Burmese women’s experiences with and perceptions of the IUD and identify avenues for improving contraceptive service delivery along the Thailand-Burma border.

**Methods:**

In the summer of 2013, we conducted in-person in-depth open-ended interviews with 31 women who obtained IUDs from a clinic along the border. We conducted a content and thematic analysis of these data using both *a priori* (pre-determined) and emergent codes and inductive techniques.

**Results:**

Women’s experiences with the IUD are overwhelmingly positive and the experiences of friends and family impact use of the device. Financial considerations and access to reproductive health facilities also shape the use of the IUD in this region. The IUD is rare along the Thailand-Burma border and misinformation about this method of contraception is pervasive.

**Conclusion:**

Our findings suggest that this modality of contraception is culturally acceptable and may be able to address structural barriers to reproductive health services along the Thailand-Burma border. Ensuring that information provided by health care providers and among peer groups is evidence-based, a full range of contraceptive methods is available, and adoption of an IUD is affordable are priorities for expanding access to reproductive health services in this setting.

## Background

For more than half a century, Burma^a^ has been characterized by severe human rights violations, civil conflict, and persecution of ethnic and linguistic minorities. The military regime surrounded villages with landmines, forced slave labour among villagers, sexually assaulted women, and burned existing crops [1,2]. Combined with inequity in food distribution, healthcare services, and education the situation has led to the displacement of millions; this includes the conflict-affected communities and internally displaced persons (IDPs) in Burma’s Eastern states as well as the approximately 1.5 million people living in Thailand as undocumented migrants or as residents of the nine “unofficial” refugee camps [1].

This overall context has had profound consequences for reproductive health. The maternal mortality ratio (MMR) in Eastern Burma is estimated at 700 to 1,200 [3,4], more than three times the national average. The contraceptive prevalence rate in Burma as a whole has long hovered at less than 40% and is estimated to be significantly less in conflict affected areas [5,6]. One of the greatest markers of unmet contraceptive need in Eastern Burma is the prevalence of unsafe abortion. Under Burmese law, abortion is only legally permitted to save the life of the woman and this legal exception is narrowly interpreted [7]. These restrictions on abortion combined with heightened unintended pregnancy risk have devastating health consequences; unsafe abortion may account for as much as 50% of maternal mortality in Eastern Burma [8].

Although the reproductive health context in Thailand is considerably less bleak than in Burma [9], women from Burma residing in Thailand also face significant barriers to accessing comprehensive services. Poverty, sexual violence and exploitation, restrictions on movement, precarious legal status, lack of trained providers, and socio-cultural taboos have resulted in considerable unmet contraceptive need and increased risk of unintended pregnancy [10]. In Thailand, abortion is legally permissible in order to protect the life, physical health, or mental health of the woman, if the pregnancy was the result of rape or incest, or if the woman became pregnant when she was 15 years of age or younger [11,12]. However, women from Burma are often unable to seek legal and safe abortion care, even if they would otherwise be eligible [10,13]. Consequently, unsafe abortion is common on both sides of the Thailand-Burma border.

Given this overall context, methods of long-acting reversible contraception (LARC), such as the intra-uterine device (IUD) or the subdermal implant, show considerable promise for improving reproductive health along the border. Safe, highly effective, and non-user dependent, LARC methods have the potential to mitigate some of the structural barriers that shape women’s consistent and timely access to contraception. However, in the face of a dearth of trained providers, an inconsistent supply of contraceptive devices, and misinformation among both providers and communities, few women on the Thailand-Burma have adopted LARC methods [10]. We undertook this qualitative study in order to shed light on women’s experiences with the IUD with the hope of informing service delivery in this long-term conflict setting.

## Methods

In the summer of 2013, we conducted in-person, in-depth, open-ended interviews with women from Burma who had received an IUD at a clinic along the border. The objectives of our study were to 1) Understand better women’s perceptions of and experiences with the IUD; 2) Explore women’s knowledge of the IUD and the ways they talk about the method with friends, family, clinicians, and others; and 3) Identify strategies for improving the quality of IUD and other contraceptive service delivery on the border and expanding access to IUDs in this region. All of our interviews took place in Tak Province, Thailand. We received approval to conduct this study by the Health Sciences and Sciences Research Ethics Board at the University of Ottawa (File #H02-13-08) as well as local research clearance from the Mae Tao Clinic (MTC), Mae Sot, Thailand.

### Study design

Women were eligible to participate in the study if they were current or past users of an IUD that they had obtained from a clinic operating along the border, had used the IUD for at least six months, were over 18 years of age at the time of the interview, and were sufficiently fluent in English, Burmese, or Karen to complete an interview. We used a multi-modal recruitment strategy that included community-based posters and flyers, announcements (with permission) at two local providing facilities, circulation of study information through the personal networks of the members of the study team, and early participant referrals (snowball sampling). Prospective participants contacted the study team by phone at which point we confirmed eligibility, provided additional information about the project, and arranged a time and location for the in-person interview.

After answering any questions and obtaining informed consent JG conducted all interviews with the aid of an interpreter (SH or HM). Using an interview guide developed specifically for this study, we began the semi-structured interview with general demographic questions before moving to questions related to the participant’s sexual, contraceptive, reproductive and pregnancy history, her experience(s) with the IUD, and her knowledge of the IUD and contraception in general. We concluded the interview with a discussion of ways in which reproductive health services could be improved along the border. We modified the initial guide in light of early interviews and participant feedback to ensure that questions were clear and culturally resonate. With participant permission, we audio-recorded interviews and took extensive field notes during and after the encounter; JG also formally memoed after each interview and regularly debriefed with other study team members. Interviews lasted an average of one hour and took place in a quiet and secure location (either at one of the clinics or at a place of the participant’s choosing). As a thank you for participating in the study and to cover any associated travel costs, each woman received 300 Thai Baht (approximately USD10) as well as snacks and drinks throughout the interview.

### Data analysis

We analyzed our data for content and themes using an inductive and iterative process [14]. The memos and regular team meetings allowed us to continuously review content and establish thematic saturation. At the conclusion of the data collection period, a member of the study team transcribed audio recordings verbatim and then translated all of the interviews into English. The content of the interviews combined with insights derived from the memos and field notes allowed for the creation of an initial code book which one member of the study team (JG) used to code all data; AF reviewed coding and disagreements were resolved through discussion. Using Atlas.TI to manage the data, we analyzed the interviews using both *a priori* (pre-determined) and emergent codes and used inductive techniques to identify themes, sub-themes and ideas. Regular team meetings aided the analytic process and the interpretation of results. This article focuses on significant findings that emerged with quotes to illustrate major themes; we have removed and/or masked all personally identifying information of participants and have used pseudonyms throughout.

## Results

### Study participants

We conducted interviews with 31 women who met our eligibility requirements. Participants averaged 32 years in age and ranged from 21 to 55 years old. Twenty eight of our participants had received their IUDs at one of two health care organizations operating on the Thai side of the Thailand-Burma border; the other three participants received their IUDs in Burma. On average, women had used the IUD for two years; duration of use ranged from 6 months to 20 years. Consistent with the specific devices available along the border, all of our participants had used or were using a Copper-T IUD. All of our participants were married at the time of the interview and, consistent with local standards of care, had obtained the IUD after getting married. Thirty of our 31 participants had at least one child at the time of the interview. At the time of the study, two of the women resided in Eastern Burma, eight of the women were migrants living in or around Mae Sot, and the remaining twenty-one were refugees from the Mae La refugee camp. Our participants reflected a range of literacy levels, educational backgrounds, and histories in the labour force.

### Women’s experiences with the IUD are overwhelmingly positive

If we use the pill, we will have a lot of side effects. If we use depo [Depo-Provera], it makes us fat. And we don’t want to be fat…Before using the IUD I had to worry all the time about whether or not I would get pregnant or when my menstruation would come…for the IUD you don’t need to remember every day. So it is really good. Mai, age 32, refugee

The overwhelming majority of our respondents spoke very highly of the IUD. Women repeatedly described the IUD as being user-friendly, effective, and long-lasting, features that women considered to be extremely positive attributes of the device. Several women also mentioned return to baseline fertility immediately after removal as an especially important consideration. As stated by Siblut, a 23 year old refugee, “When you take out the IUD, you have a chance to get pregnant [again].”

Many of our participants had used hormonal methods of contraception at some point during their reproductive lives. According to the women we interviewed, commonly available hormonal contraceptives, such as oral contraceptive pills and Depo-Provera, resulted in side effects, including weight gain, dizziness, and irregular bleeding patterns. That women experience few side effects with the IUD was one of its most positive attributes.

If we use the IUD, menstruation comes every month so we don’t have many side effects. But if we use other things, we have side effects…we get very fat or [have] muscle tension. [For us] the IUD is good and not bad. Yon, age 28, refugee

This is not to say that all aspects of the IUD were well received. Many of our participants reported that they had experienced pain and cramping in the first few days after insertion but felt prepared for these side effects because of the counseling they had received from clinicians. Women were especially appreciative of the quality of care provided by organizations on the Thai side of the border that serve Burmese populations. Further, some of the women who had experienced labor and delivery explained that the pain during childbirth is much more severe than the pain during an IUD insertion, thus putting pain and discomfort in a broader context of their reproductive health histories.

Few women that we spoke with experienced significant side effects or complications; of the 31 women interviewed, one woman experienced contraceptive failure and became pregnant while using the IUD, one woman had her IUD removed after two years because of a “bad odor,” and one woman had her IUD removed because of persistent abdominal pain. In the latter two cases, the participants were unsure as to whether or not the IUD was the primary cause of the symptom. However, all three of these women still spoke highly of the IUD and recommended the device to women in their communities.

### Financial considerations and access to health facilities shape use

I think the IUD is good. We don’t need to take it, like the depo, every 3 months. And even when we are putting the IUD in, there is no frustration. We don’t need to worry about anything. If we are using depo, we have to go back every 3 months, and also when we are getting depo, it is painful to get every time. Eh Say Gay, age 32, refugee

In a setting with high unemployment and considerable poverty, the financial implications of unintended pregnancy emerged as a primary theme. Nearly all of our participants noted that use of a highly effective method of contraception was of paramount importance. As stated by Thaw Thaw, a 19 year old migrant living in Mae Sot, “[I decided to use] contraception because we both have no work…if we have kids and we have no job, our kids will be in trouble.” That IUDs are understood by users to be more effective than other non-permanent methods of contraception was critical. “If I didn’t use the IUD, I would have had more than 10 children by now” (Myia, age 54, IDP). Many women talked to their husbands about the IUD prior to insertion and the majority reported that their husbands were supportive of the decision.

Women also referenced that use of the IUD eliminates the need for regular visits to a health care provider for contraception. For women living outside of refugee camps, this quality was especially important, as undocumented migrants face considerable barriers to accessing clinics, including the threat of deportation, arrest, and/or fines if caught by authorities while in transit. One participant discussed in detail the challenges that undocumented migrants face, most of whom work long and inflexible hours, in obtaining health services.

We get one day off on Sunday and we have regular working hours. We usually start at 8 am and work until 10 pm. Sometimes they even keep us working until 12, midnight… I live with my husband in the factory and sometimes we send money to mother [in Burma], as much as we have…[Before this job] I didn’t have stable work or school because I’m the oldest daughter and have to support my family…sometimes I just work here [in Mae Sot] as a nanny, and sometimes I work in construction. So I have experience in odd jobs….I suffer from urine pain very often and I cannot take time off from work at the factory to go to the clinic… We simply cannot take a leave from work.

Lwin, age 22, migrant

### Experiences of friends and family impact use

I [heard] some people say that if you use the IUD it can cause cancer in our uterus and I was scared to use it…But my sister, she explained that she used the IUD and that she was feeling good with no side effects, that’s why I started using it and now I feel good! Kyaw, age 35, refugee

Women living along the border rely heavily on the opinions and experiences of their friends and family when making reproductive health decisions. Throughout the study, women consistently reported that their main sources of contraception information came from their close social networks. Indeed, most participants presented at the clinic having already made the decision to adopt an IUD. As Eh Htoo Ne (age 28, refugee) explained, “I didn’t know anything about this [the IUD]. But because my friends suggested to me that this is good, I went [to the clinic] and got it inserted.” Exceptionally, the small number of our participants who were trained in health services had generally been exposed to the IUD through their formal education, in-service training, or work.

After adopting the IUD, many women in our study became resources in their own circles, sharing their experiences with friends and family. Participants repeatedly shared stories about how they answered women’s questions and helped spread awareness of the method.

Whenever I meet with young women, I say “you know, you have never used the IUD, that’s why you have more and more children. [I understand that] you are scared of putting in the IUD. But you can see that we have put in the IUD and we have stayed healthy. I have never heard of anyone putting in the IUD and dying. So you can put it in and not die…Here [in Thailand] you can put in the IUD for free…you don’t have to worry about that.

Myia, age 54, IDP

### Use of the IUD along the border is rare

In Burma it is really rare that [women] use the IUD, and also it is expensive; they don’t provide it in hospitals. So if you want to use the IUD, you have to search for a clinic that provides it. Wahksha, age 36, refugee

Despite the enthusiasm for the IUD among users, our participants reported that adoption of the IUD was extremely rare. Women explained that knowledge of the IUD is virtually non-existent in Eastern Burma and most women only learned about the IUD once arriving in Thailand. In addition to the high cost associated with this contraceptive method, women explained that in Burma it was difficult to find a reliable and accessible clinic that offered the IUD. In stark contrast with the situation in Burma, several facilities operating on the Thai side of the border provide a full range of non-permanent methods of contraception at low or no cost. Lwin (age 22, migrant) expressed her appreciation of these services, “But here [in Thailand], even if you have no money, they [the Mae Tao Clinic] just provides a free service for us.”

Although knowledge of the IUD is perceived as being low, our participants reported that negative rumors about the device are rampant. As explained by women in our study, popular misconceptions include beliefs that the IUD causes cancer or ectopic pregnancy, the device is not effective in preventing pregnancy, and husbands do not like it. Further, a commonly cited rumor is that the device “wanders” throughout the body. As reported by Daylya, a 25 year old migrant residing in Mae Sot, “They say that [the IUD] can move around the body through to the brain and arms.” Many of our participants reported that they actively engaged with women in the community to combat these rumors, correct misinformation, and allay fears.

### More education and awareness about the IUD is needed

It would be better if we were able to enter the community and offer education about…reproductive health and also about contraception and explain the types of contraception…like the IUD; how it works, the negatives and positives…stuff like that. Wahksha, age 36, refugee

Nearly all of our participants discussed the importance of raising awareness about family planning in the community. The majority explicitly stated that these educational efforts should be targeted to both married and unmarried women, as comprehensive reproductive health services for unmarried women are minimal. Some women suggested that holding group discussions to provide information about the IUD and debunk IUD-related myths would be an effective community-based awareness raising strategy. Other women discussed how clinics could do a better job of “advertising” the IUD though posters with a picture and other patient education materials. Many participants referenced similar efforts that are currently underway that focus on other methods of contraception and suggested that this could serve as a model for comparable efforts related to the IUD.

## Discussion

Until recently, few efforts have been undertaken to expand access to the IUD along the Thailand-Burma border [10,15]. The limited published literature indicates that the IUD is not widely used in Burma due to a lack of trained providers and misinformation among both health care workers and communities [3,10,16]. Our findings are broadly consistent with this larger body of work. However, no published work has explored women’s experiences with the IUD and our study aimed to fill this gap.

That users of the IUD on the Thailand-Burma were overwhelmingly positive about their experiences suggests this modality of contraception has cultural and social resonance and may serve as an important addition to the mix of family planning methods that are currently available. Indeed, women’s identification of effectiveness, ease and longevity of use, and safety as important features of the IUD is consistent with the global literature on acceptability and adoption [17-19]. The socio-cultural acceptability of the IUD (for use among married women) is also signaled by the supportive response women received from their husbands once making the decision to adopt this method.

Yet the reality of women’s lives on the Thailand-Burma border may make the IUD an especially valuable addition to the existing methods mix. In this protracted conflict setting where women’s freedom of movement is limited and fear of arrest and deportation abounds [1,20], a reversible contraceptive that only requires an initial visit to a trained provider has the potential to address significant structural barriers that impede many women’s ability to contracept most effectively [10,20]. Further, in a setting where abortion is severely legally restricted (Burma) and largely inaccessible to women from Burma (Thailand), the consequences of an unwanted pregnancy can be dire; unsafe abortion carries a significant risk of reproductive morbidity and carrying an unwanted pregnancy to term and parenting can place families in a precarious financial position. That our participants identified the IUD as serving a mitigating role in addressing structural obstacles to pregnancy prevention suggests its utility in this setting.

Yet few women appear to be using the IUD and misinformation abounds. Consistent with the global literature [17,19,21,22], pervasive myths about the IUD include the belief that the device increases the risk of cancer and ectopic pregnancy, moves around the body, and is ineffective at preventing pregnancy. That the IUD is inserted and is therefore hidden from sight fosters imagined and exaggerated medical risks. A study published by Russo et al. highlights and corrects the many myths and misconceptions found in the literature surrounding LARC methods [23]. Creation of culturally and linguistically tailored information about the IUD that could serve to combat myths in this setting appears warranted.

However, our findings suggest that it is women within the community that may have the most significant impact on social norms and perceptions of the IUD. Discussions about contraception among friends and family members had a profound impact on the women we interviewed and appeared to be more influential than counseling by health service providers or the values expressed by (largely male) religious leaders or community elders. This finding is again consistent with a global body of research dedicated to reproductive health decision-making [24-27]. Our findings suggest that identifying avenues for dissemination of evidence-based information through peer networks could be especially impactful in this context.

Our findings point to several avenues for improving contraceptive service delivery along the border. Doctors and clinicians can play a significant role in the administration of the IUD and the community’s perception of the method. By understanding users’ perceptions of and experiences with the IUD, medical workers will be in a better position to respond to questions and provide tailored and targeted counseling to women seeking family planning or post-abortion care services. Further, since many women rely on family and friends’ opinions and advice, clinics can take advantage of women’s perceptions of the device to raise awareness about the method. Heeding the suggestions provided by our participants and supporting community-based educational workshops and user-friendly patient education materials about the IUD should be prioritized.

The results from this study have shed light on the features of the IUD that are the most salient with women living on the Thailand-Burma border. Taken together, the experiences of women who have used the IUD and their recommendations for how to address the misinformation about the IUD that is pervasive among women and members of their communities offer health service providers insights into the ways in which contraceptive information, counseling, and service delivery could be improved.

### Limitations

As is true with qualitative research in general, the findings of this study cannot be generalized to the population living on the Thailand-Burma border. The majority of our participants lived in or around Mae Sot, Thailand and the Mae La refugee camp and we only conducted the study in English, Burmese, and Karen, thus the diversity of our participants was limited. Further, by only including women who had used the IUD for at least six months we did not capture the experiences of women who requested removal soon after insertion. Finally, at the time of our study, hormonal implants were not generally available along the border and thus we limited our investigation to only one LARC method. However, we are confident that the themes that we identified are significant and that the credibility and trustworthiness of the findings were enhanced by our multi-disciplinary, multi-lingual study team.

## Conclusion

Women’s experiences with the IUD suggest that this modality of contraception is culturally acceptable and may be able to address structural barriers to accessing reproductive health services along the Thailand-Burma border. Ensuring that information provided by health care providers and among peer groups is evidence-based, a full range of contraceptive methods is available, and adoption of an IUD is affordable are priorities for expanding access to reproductive health services along the Thailand-Burma border. The lessons learned from this project provide insight into women’s experiences on the Thailand-Burma border and may suggest avenues for improving reproductive health services in other protracted conflict and refugee settings.

## Endnote

^a^The name “Myanmar” was determined by the military junta in 1989. Use of “Myanmar” is perceived by many ethnic minority groups and organizations operating along the border as legitimizing the ruling military and its authority to rename the country. Out of respect for local stakeholders and to reflect the language used by women who participated in the study, we will refer to the country as “Burma” throughout this article.

## Abbreviations

IDP: Internally displaced person
IUD: Intra-uterine device
LARC: Long-acting reversible contraception
MMR: Maternal mortality ratio
MTC: Mae Tao Clinic
